# Comparative Analysis of Alternative Splicing in Two Contrasting Apple Cultivars Defense against *Alternaria alternata* Apple Pathotype Infection

**DOI:** 10.3390/ijms232214202

**Published:** 2022-11-17

**Authors:** Tingting Zhou, Youlei He, Xianqi Zeng, Binhua Cai, Shenchun Qu, Sanhong Wang

**Affiliations:** College of Horticulture, Nanjing Agricultural University, Nanjing 210095, China

**Keywords:** apple, alternative splicing, *A. alternata* AP, RNA-Seq, post-transcriptional regulation

## Abstract

Alternaria blotch disease, caused by the *Alternaria alternata* apple pathotype (*A. alternata* AP), is one of the most serious fungal diseases in apples. Alternative splicing (AS), one of the pivotal post-transcriptional regulatory mechanisms, plays essential roles in various disease resistance responses. Here, we performed RNA-Seq for two apple cultivars (resistant cultivar ‘Jonathan’ (J) and susceptible cultivar ‘Starking Delicious’ (SD)) infected by *A. alternata* AP to further investigate their AS divergence. In total, 1454, 1780, 1367 and 1698 specifically regulated differential alternative splicing (DAS) events were detected in J36, J72, SD36 and SD72 groups, respectively. Retained intron (RI) was the dominant AS pattern. Conformably, 642, 764, 585 and 742 uniquely regulated differentially spliced genes (DSGs) were found during *A. alternata* AP infection. Comparative analysis of AS genes in differential splicing and expression levels suggested that only a small proportion of DSGs overlapped with differentially expressed genes (DEGs). Gene ontology (GO) enrichment analysis demonstrated that the DSGs were significantly enriched at multiple levels of gene expression regulation. Briefly, the specific AS was triggered in apple defense against *A. alternata* AP. Therefore, this study facilitates our understanding on the roles of AS regulation in response to *A. alternata* AP infection in apples.

## 1. Introduction

The apple (*Malus* × *domestica* Borkh.) is one of the most important economic fruits around the world, while severely limited by various fungal diseases [1,2]. Alternaria blotch disease caused by the necrotrophic pathogen, *Alternaria alternata* apple pathotype (*A. alternata* AP), is one of the most serious fungal diseases in East Asia affecting apple quality and yield via damaging young shoots, leaves and fruits, leading to huge economic losses [3]. Previously, most studies have mainly focused on *A. alternata* pathogenicity and toxicity [4,5,6], while only a few report on apple–pathogen interactions [7,8,9]. It indicated that the different resistance of apple varieties to *A. alternata* AP was associated with the expression divergence of pathogenesis-related (PR) proteins and some defense-related proteins [7]. Another report suggested that plant growth regulators could vary the resistance to *A. alternata* AP via modulating signaling and metabolic networks in apples [10]. Our previous studies showed that the downregulation of PR genes and cell wall defensive vulnerability increased ‘Starking Delicious’ susceptibility to *A. alternata* AP [8]; the increase and accumulation of thaumatin-like protein (PR5), endochitinase and (+)-neomenthol dehydrogenase at early infection stage granted the ‘Jonathan’ ability to contend *A. alternata* AP invasion [9].

Alternative splicing (AS) is a process of generating multiple transcripts during the transcription of a single precursor mRNA in eukaryotes due to the existence of different splicing sites, which increases the diversity of transcripts and proteins, modulates gene expression through the post-transcriptional regulatory mechanism and plays crucial roles in planta growth, development and various stress conditions [11,12]. Major types of AS events include skipped exon (SE), retained intron (RI), alternative 3′ splice sites (A3SS), alternative 5′ splice sites (A5SS) and mutually exclusive exon (MXE) [13,14]. The ratios of different type events are distinctive in animals and plants; skipped exon is the prevalent event in animals [15,16], while retained intron is the predominant event in multiple mode plants [17,18,19].

So far, some progress on the research of alternative splicing in plants has been made. For instance, the AS profiles of various plants have been reported, such as Arabidopsis [20], soybean [21], rice [22,23], tomato [24], wheat [25], maize [26] and so on. Extensive examples uncover that the AS widely involves in plant development and stress responses, including seed germination [27], root development [28], shoot growth [29], flowering transition [30], fruit maturation [31], cold [32,33], heat [34], salt [35,36], drought [37]; and flooding stress [38], hormone signaling [39,40,41] and nutrient deficiency [42,43].

Evidence is accumulating that the AS also plays important roles in plant–pathogen interaction [44,45,46]. A previous study performed transcriptome analysis on the pea (*Pisum sativum* L.) roots in symbiosis with arbuscular mycorrhiza (*Rhizophagus irregularis*), establishing the feedback loops of gene expression during mycorrhization [47]. Besides, gene co-expression network among differentially spliced genes showed that WRKY, NAC, bHLH, AP2/ERF-ERF, etc., families were the major modulators in rapeseed (*Brassica napus*) defense against *Leptosphaeria maculans* [48]. A paradigm of wheat (*Triticum aestivum* L.) inoculated with powdery mildew, *Blumeria graminis* f. sp. *tritici* (*Bgt*) and stripe rust fungus, *Puccinia striiformis* f. sp. *tritici* (*Pst*) demonstrated that both disease resistance proteins and splicing factors could undergo AS changes; additionally, specific splice transcripts were induced or activated to defense against two fungal pathogens [49]. Further experimental study elucidates that OsWRKY62.1 and OsWRKY76.1 negatively regulate the rice resistance to the blast fungus *Magnaporthe oryzae* (*M. oryzae*) and the leaf blight bacterium *Xanthomonas oryzae* pv *oryzae* (*Xoo*), in addition, two truncated OsWRKY62.2 and OsWRKY76.2 proteins exhibit reduced binding to W box motif illustrating the negative feedback regulation of WRKY proteins during pathogen defense stage in rice [50]. *Phytophthora infestans* effector *SRE3* mediates tomato AS mechanism and thereby subverts plant immunity [12]. Interestingly, the splicing patterns of *PR3b* in the low-nicotine mutants of ‘Burley 21’ tobacco suggesting the potential mechanism of the ET/JA signaling pathway modulating plant immunity [51]. Prior evidence indicated that the alternative splicing isoforms of apple *NPR1* homologs may contribute to disease resistance [41]. Furthermore, osa-miR7695 targeting an AS transcript of *OsNramp6* regulates the blast fungus *M. oryzae* resistance, manifesting the existence of a combined regulatory network through which miRNA function and AS regulation during fungal infection are integrated [52].

However, the AS landscape in apple response to *A. alternata* AP infection has been unclear and the mechanism of AS changes triggered by pathogen infection needed to be further explored. In this study, we constructed sixteen RNA-Seq libraries and sequenced with Illumina HiSeq platform using the apple leaves of resistant cultivar ‘Jonathan’ and susceptible cultivar ‘Starking Delicious’ inoculated with *A. alternata* AP pathogen, aimed at investigating the role of AS in apple resistance to *A. alternata* AP. We found AS divergence in the two cultivars at different periods of pathogen infection. This study helped to understand the disease mechanism of an apple against *A. alternata* AP.

## 2. Results

### 2.1. General Analysis of RNA-Seq Data

RNA-Seq was applied to characterize AS events in leaves of resistant cultivar ‘Jonathan’ and susceptible cultivar ‘Starking Delicious’ at 36 and 72 h post inoculation (HPI) with *A. alternata* AP, with samples in mock-inoculation conditions (hereafter named CK) as controls. In total, an average of 44.54 million (44,536,243) clean reads were obtained from all the samples with an average of Q20 up to 97.90%. Additionally, an average of 35.23 million (35,233,324) mapped clean reads approximately occupying 79.11% of clean reads, were utilized to assemble into transcripts (also called gene isoforms) using Cufflinks software (2.2.1), about 99.80% of them mapped unique location on the apple reference genome (Table S1).

**Figure 1:**
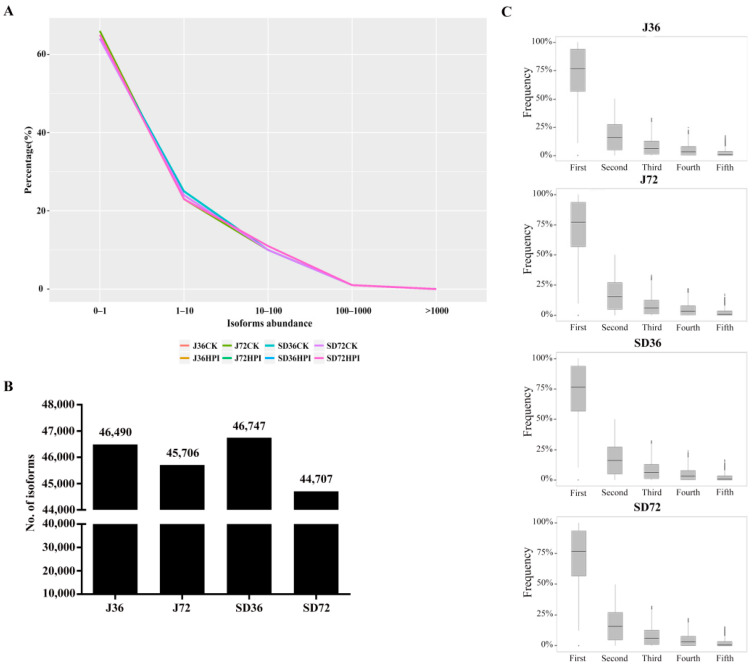
The number and abundance distribution of isoforms in apple. (**A**) The distribution of isoforms abundance in eight samples overlapped with two biological replicates. The isoforms abundance was calculated with FPKM. (**B**) The number of isoforms in J36, J72, SD36, SD72 groups. (**C**) Frequency of the top five most abundant isoforms in each gene across J36, J72, SD36, SD72 groups.

Expression profiles of all predicted isoforms from eight overlapped transcript datasets (J36CK, J36HPI, J72CK, J72HPI, SD36CK, SD36HPI, SD72CK and SD72HPI) by merging the two biological repeats revealed that the expression abundance of over 60% of the isoforms below 1 FPKM, approximately 25% between 1 and 10 FPKM, and about 10% higher than 10 FPKM (Figure 1A). It has been reported that isoforms with expression levels lower than 1 FPKM could not be translated into functional proteins [53], meaning that most isoforms might play a regulatory role at the transcriptional or post-transcriptional level. Currently, sixteen sample libraries were divided into four comparative groups: J36CK vs. J36HPI (J36), J72CK vs. J72HPI (J72), SD36CK vs. SD36HPI (SD36), SD72CK vs. SD72HPI (SD72). According to the previous research [53], we defined the isoforms with a threshold FPKM >1 as effectively expressed isoforms and screened them for further analysis. Altogether, 59,735 effectively expressed isoforms corresponding 28,152 genes were obtained; specifically, 46,490 isoforms were detected in the J36 group, 45,706 in J72, 46,747 in SD36, and 44,704 in SD72, respectively (Figure 1B). Given the isoforms in the annotated loci, a large number of the splicing junctions (SJs) resides in the coding sequence (CDS), manifesting that the potential influence of AS on protein products (Figure S1A). In addition, sequence analysis of the splicing donor–acceptor sites for all expressed isoforms confirmed that GT-AG splice pairs accounted for a dominant percent (~98%), followed by GC-AG, and the remaining type occupied less than 1% (Figure S1B). Additionally, nearly 50% of these multi-exon genes had more than one isoform as a result of multiple AS events occurred in one gene (Figure S1C). After removing one isoform gene, we further calculated the proportions of the top five expressed isoforms among multi-isoform genes. The result showed that the abundance of top1 and top2 isoforms accounted for about 75% and 20% of total isoforms in each comparative group, respectively (Figure 1C). Apparently, AS contributed to the production of genes with multi-isoforms. Therefore, we further depicted the AS landscape in apple transcriptome.

### 2.2. Detection and Classification of AS Events in Responding to A. alternata AP Infection in Apples

**Figure 2:**
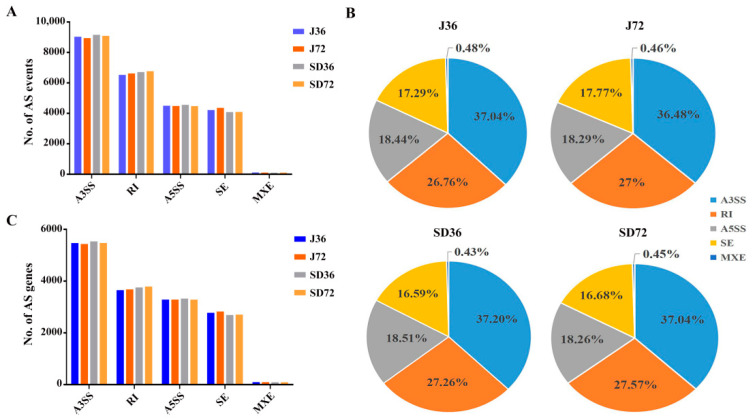
Summary of AS events and genes in apple. (**A**) The number of five AS events in response to *A. alternata* AP infection in apples. Color scale represents J36, J72, SD36 and SD72 groups, respectively. (**B**) The distribution of five AS events in J36, J72, SD36 and SD72 groups. Color scale represents A3SS, RI, A5SS, SE and MXE AS event types, respectively. (**C**) The number of five AS genes in response to *A. alternata* AP infection in apples. Color scale represents J36, J72, SD36 and SD72 groups, respectively.

To better understand the impact of pathogen infection on alternative splicing regulation, we applied the rMATS program to examine the five major AS events including RI, SE, A3SS, A5SS and MXE (Figure 2). Here, a total of 34,753 AS events were identified in all four groups. These AS events were generated from 11,421 genes, accounting for 40.15% of all expressed genes, with an average of 3.04 AS events per gene. In detail, A3SS events (13,084, 37.65%) were the most predominant type, followed by RI (9004, 25.91%), A5SS (6398, 18.41%), SE (6125, 17.62%) and MXE (142, 0.41%). Not surprisingly, the proportion of five different AS types in four comparative groups were consistent with total events presented above (Figure 2A,B and Table S2). It’s noteworthy that A3SS, RI, A5SS, SE and MXE five type AS events were distributed in 7073 (61.93%), 4640 (40.63%), 4353 (38.11%), 3793 (33.21%), and 117 (1.02%) genes, respectively, which also exhibited similar distribution in four comparative groups (Figure 2C and Table S3).

In addition, the distribution of the length of different AS events displayed significant differences. For SE, the skipped exon length ranged from 4 bp to 3681 bp, with the length of 75 bp as the largest number of events. However, for A3SS and A5SS, the lengths of 3 bp and 4 bp were the peak distributions, respectively. The RI events length distribution concentrated in the range from 299 bp to 813 bp, with two peaks at 606 bp and 637 bp (Figure S2A). We also found the length of intron in RI events was significantly longer than the average length of intron in the total genome, while the length of exon in SE events was shorter than the average length of exon in the total genome (Figure S2B). Furthermore, we subdivided the length of each event into three categories, i.e., the number of nucleotides in the splicing events is divided by three, if the remainder is zero it is named AS0, if the remainder is one it is named AS1, and the remainder of two as AS2. AS1 and AS2 might introduce premature stop codons (PTC) and lead to the production of short, truncated proteins, but AS0 does not. Strikingly, the AS1 and AS2 type occupied 64.05~70.23% in five AS events, intron and exon in the total genome, while the AS0 type only occupied 29.77~35.95%. Nevertheless, the ratio of AS0 in A3SS (34.4%) and A5SS (35.95%) higher than that in intron (33.45%). Alongside, the ratio of AS0 in SE (31.12%) and MXE (34.51%) were higher than that in exon (29.77%) (Figure S2C). Therefore, AS events in apples withstand evolutionary pressure to maintain the open reading frame (ORF).

**Figure 3:**
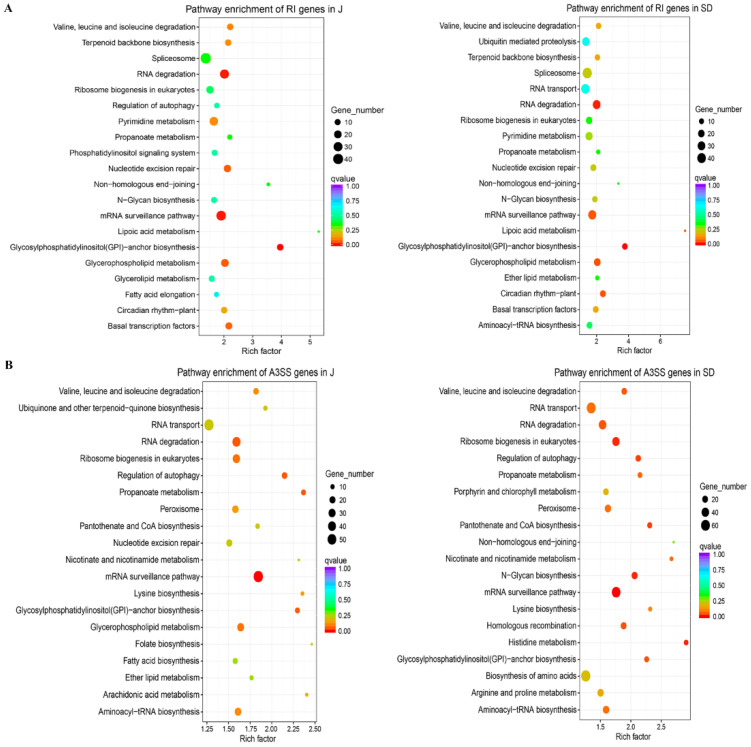
Comparative analysis of enriched KEGG pathways of RI and A3SS genes between J and SD in response to *A. alternata* AP infection. (**A**) Pathway enrichment of RI genes of J and SD in response to *A. alternata* AP infection. (**B**) Pathway enrichment of A3SS genes of J and SD in response to *A. alternata* AP infection. J: overlapped J36 and J72 groups; SD: overlapped SD36 and SD72 groups. The adjusted-*p* values are given with the color bar. The rich factor is noted at the x-axis and gene numbers are indicated by bubble scale.

Moreover, a comparison of represented Kyoto Encyclopedia of Genes and Genomes (KEGG) pathways among five types of AS genes between J and SD cultivars was performed using KOBAS v2.0 in the R software package (Figure 3). For RI genes, ‘mRNA surveillance pathway’ was also significantly enriched in J cultivar, in addition to pathways of ‘glycosylphosphatidylinositol (GPI)-anchor biosynthesis’ and ‘RNA degradation’ in J and SD cultivars (Figure 3A). For A3SS genes, other pathways such as ‘ribosome biogenesis in eukaryotes’, ‘histidine metabolism’ and ‘N-Glycan biosynthesis’ were also remarkably enriched in SD cultivar, in addition to ‘mRNA surveillance pathway’ in J and SD cultivars (Figure 3B). In further addition to these, ‘spliceosome’ and ‘circadian rhythm—plant’ pathways were mainly enriched in SE and A5SS genes in both cultivars, respectively, whereas no represented pathway was observed in MXE genes, due to the limitation of gene number (Figure S3). These results imply that different types of AS genes are involved in different regulatory functions.

### 2.3. Identification and Analysis of DAS Events and Genes in Responding to A. alternata AP Infection

**Figure 4:**
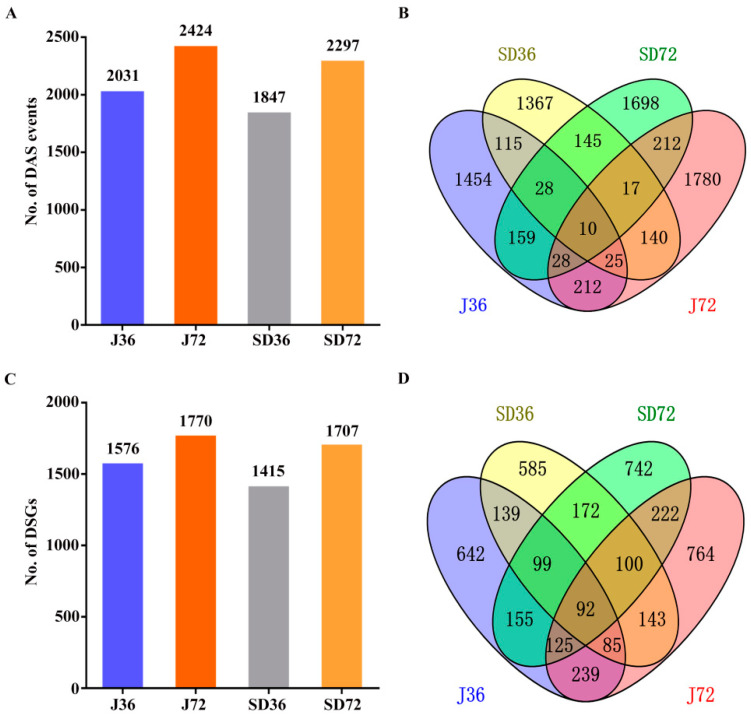
Identification and comparison analysis of DAS events and DSGs in response to *A. alternata* AP infection in apples. (**A**) The number of DAS events in response to *A. alternata* AP infection in apples. (**B**) Venn diagram showing the comparison of DAS events in response to *A. alternata* AP infection in apples. (**C**) The number of DSGs in response to *A. alternata* AP infection in apples. (**D**) Venn diagram showing the comparison of DSGs in response to *A. alternata* AP infection in apples.

To further investigate pathogen-responsive AS divergence, identification of DAS events was performed during apple leaves’ response to *A. alternata* AP infection (Figure 4). It is remarkable that there were in total 7390 DAS events were identified, including 2031 in J36, 2424 in J72, 1847 in SD36 and 2297 in SD72 (Figure 4A). In detail, comparison of DAS events suggested that 1454, 1780, 1367 and 1698 DAS events were specifically induced in J36, J72, SD36 and SD72 groups, respectively, while only 10 DAS events were shared in all groups (Figure 4B). It was apparent that the most events were cultivar or infection course-specific. AS responses were triggered intensively in J cultivar, especially at 72 HPI. Markedly differing with total AS events, RI was the most dominant type in DAS events (Table S4). Moreover, the expression of DAS events presented distinct pattern in responding to *A. alternata* AP infection, e.g., upregulated in one group and downregulated in another group (Figure S4). The above results indicate that AS involves in apples response to *A. alternata* AP infection, and exhibits notable divergence in two cultivars.

**Figure 5:**
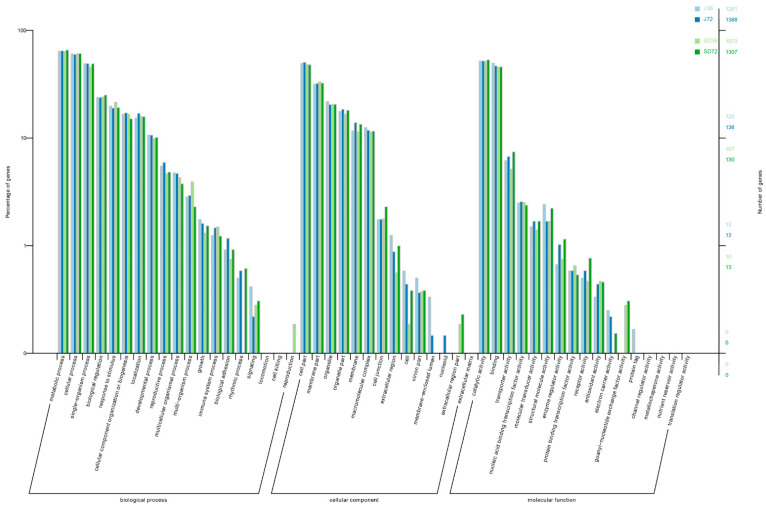
Distribution of GO terms among DSGs in response to *A. alternata* AP infection in apples. The left y-axis represents the percentage of the DSGs enriched to the GO terms. The right Y-axis represents the numbers of the DSGs enriched to the GO terms. Color scale represents J36, J72, SD36 and SD72 groups, respectively.

Correspondingly, a total of 4303 DSGs were characterized, including 1576 in J36, 1770 in J72, 1415 in SD36 and 1707 in SD72, respectively. Venn diagram showed that 642, 764, 585 and 742 DSGs were specific in J36, J72, SD36 and SD72, respectively, whereas fewer DSGs were overlapped and only 92 of them were common in all four groups (Figure 4C,D). Furthermore, the GO enrichment analysis among the DSGs displayed that 50 GO terms were overrepresented during *A. alternata* AP infection, including 19 biological processes, 14 cellular components and 17 molecular functions. In terms of these, ‘metabolic process’, ‘cell part’ and ‘catalytic activity’ were the most represented terms in the three main categories, respectively (Figure 5). Conjecturally, AS engages in various processes and exerts specific functions defense against *A. alternata* AP infection in apples.

### 2.4. Validation of AS Events in Responding to A. alternata AP Infection

**Figure 6:**
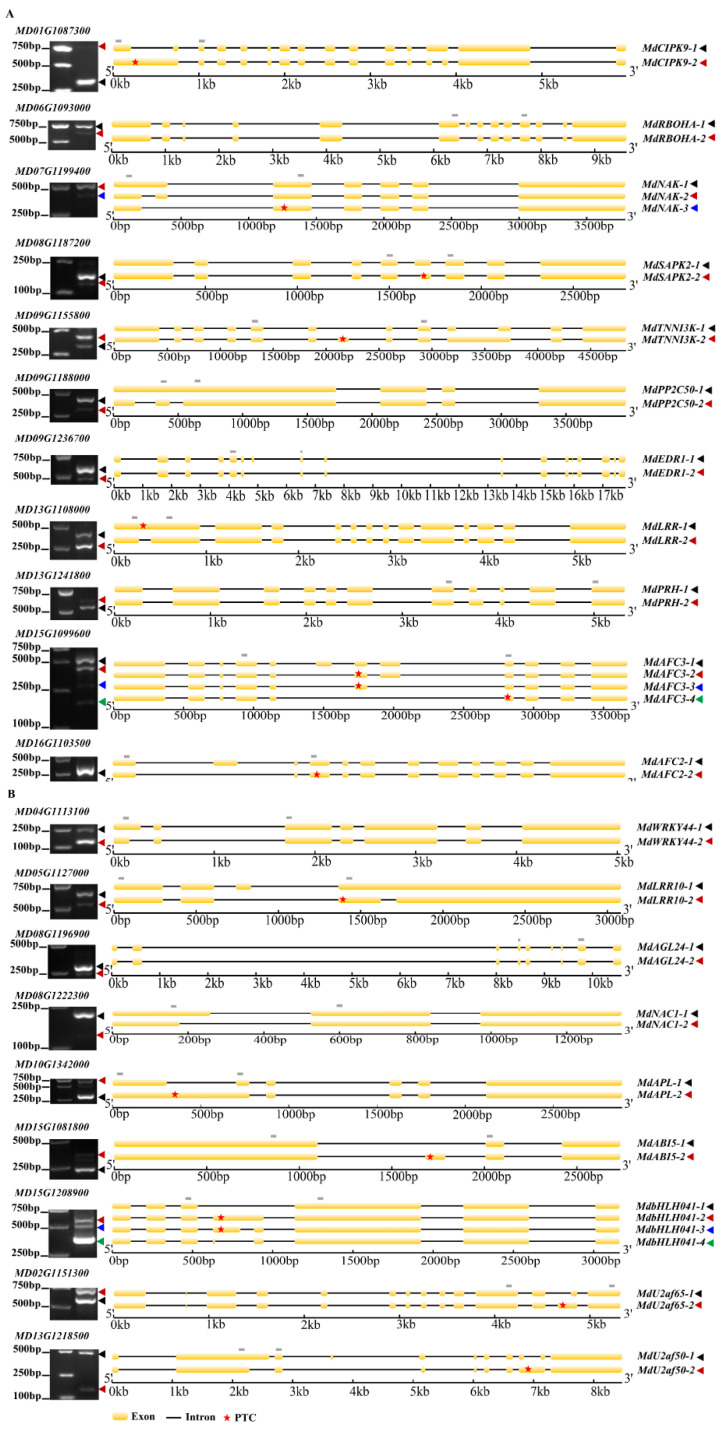
Validation of AS events by RT-PCR in response to *A. alternata* AP infection in apple. (**A**) The AS events in eleven functionally characterized genes. (**B**) The AS events in nine transcription factor and splicing factor genes. The locations of RT-PCR primers were marked with gray rectangles. The annotated and novel transcripts were marked with black and other colored arrowheads, respectively. The positions of PTCs were marked with red asterisks.

To verify the reliability of predicted AS events, transcripts of twenty AS genes, encoding several disease resistance proteins, such as serine/threonine protein kinases, many kinds of transcription factors (WRKY, NAC, MYB, bHLH, ABI etc.) and splicing factors, were validated by RT-PCR. RNA was extracted by mixing all samples. The primers spanning the AS sites of each transcript were designed (Table S5). Noticeably, eleven SE, five RI, five A5SS and two A3SS events were identified, which were highly consistent with our RNA-Seq data (Figure 6). The general information of validated AS genes was shown in Table S6. These results suggested that AS exists to a widespread degree in apple response to *A. alternata* AP infection.

For *MdPP2C50* (*MD09G1188000*), RI was found in the 5′-UTR region, which might affect mRNA translation efficiency, and then affect protein expression level although not change the integrity of the CDS region [54,55] (Figure 6A). For *MdAFC3* (*MD15G1099600*), three SE events occurred with a length of 115 bp, 263 bp and 355 bp, respectively, owing to alternative skipping of multiple exons. In addition, RI and A5SS events occurred in *MdbHLH041* (*MD15G1208900*) due to the second intron being completely or partially retained. Interestingly, SE events in *MdRBOHA* (*MD06G1093000*), *MdEDR1* (*MD09G1236700*) and *MdAGL24* (*MD08G1196900*), caused the partial deletion of peptide sequences, as well as A5SS events in *MdPRH* (*MD13G1241800*) and *MdNAC1* (*MD08G1222300*) causing the elongation of peptide sequences, which potentially affected their functions (Figure 6A,B). Sequence analysis indicated that a PTC was observed among most of the AS transcripts, which led to the production of truncated proteins with the lack of conserved domains (Figure 6 and Figure S5). Thence, these transcripts resulted in the structural and functional diversity of proteins, although they could be rapidly degraded through the nonsense-mediated-decay (NMD) pathway.

**Figure 7:**
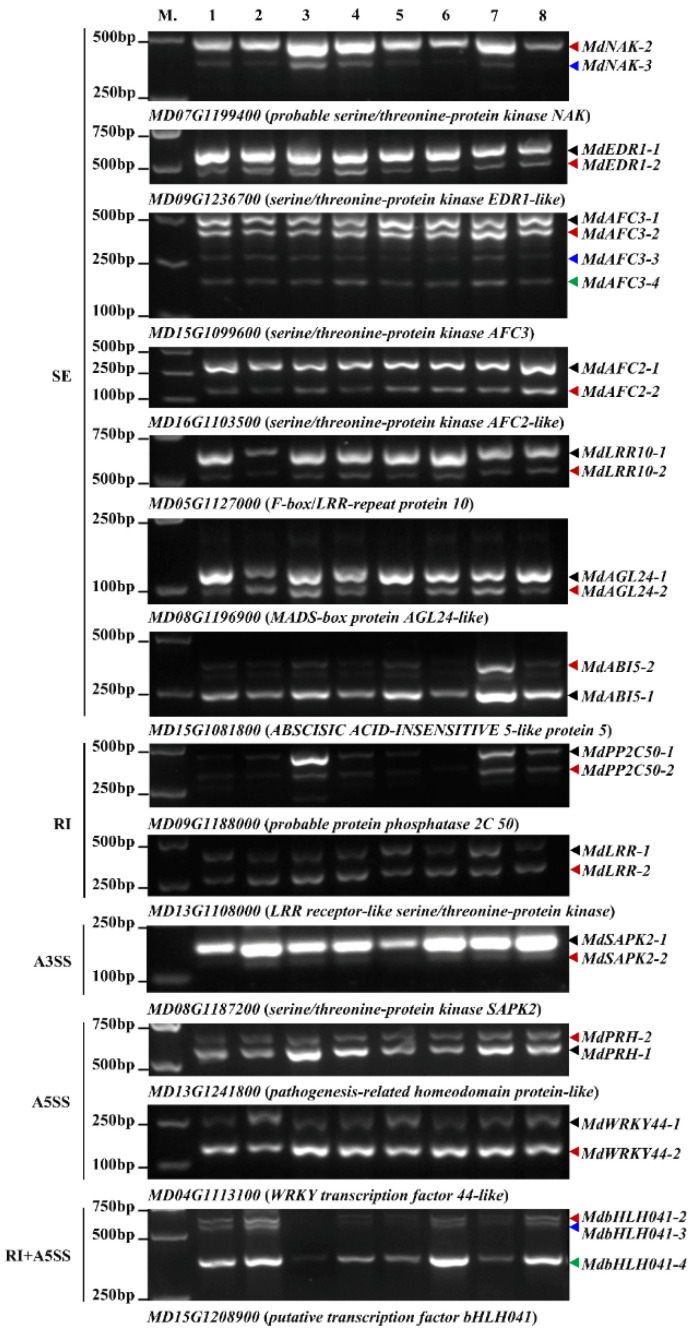
Expression patterns of pathogen-responsive AS events were validated by RT-PCR in apple leaves. Thirteen AS genes were analyzed to verify the reliability of our RNA-Seq data. Lane1 (M.) indicates the marker DL2000 and the ladder is noted on the left of agarose gel. Lane2~Lane9 (1~8) indicate the length and abundance of PCR products used to validate AS events under J36CK, J36HPI, J72CK, J72HPI, SD36CK, SD36HPI, SD72CK and SD72HPI conditions, respectively. The gene ID and annotation were given at the bottom of each panel.

Subsequently, the expression patterns of AS events were validated by RT-PCR, which were highly concordant with RNA-Seq except some differences (Figure 7 and Figure S6). Interestingly, some AS events exhibited distinct expression patterns in response to *A. alternata* AP infection. Compared to the mock-inoculation condition, the expression of one SE event in *MdAFC3-4* was upregulated in the J cultivar at 72 HPI, but downregulated in SD cultivar. In addition, one SE event of the *MdAFC2-2* transcript was downregulated in the J cultivar at both 36 and 72 HPI, whereas it was upregulated in the SD cultivar. Likewise, the expression of one A5SS event in *MdWRKY44-1* was upregulated in the J cultivar at 36 HPI, while reduced in the SD cultivar. These results indicate that the specific expression pattern of the AS events in the two cultivars was caused by *A. alternata* AP infection. However, some AS events were inhibited by *A. alternata* AP infection. For examples, an RI event in *MdPP2C50-1*, and two SE events in *MdNAK-3* and *MdABI5-1* were induced under mock-inoculation conditions, but rarely expressed under infection conditions. Intriguingly, the expression of *MdAGL24-2* was increased at 36 HPI and decreased at 72 HPI in both cultivars; moreover, *MdAGL24-1* and *MdAGL24-2* showed completely opposite expression patterns in response to *A. alternata* AP infection. Therefore, AS participates in the regulation of apple–pathogen interaction.

### 2.5. Comparative Analysis of the AS Genes in Responding to A. alternata AP Infection at AS and Transcriptional Levels

**Figure 8:**
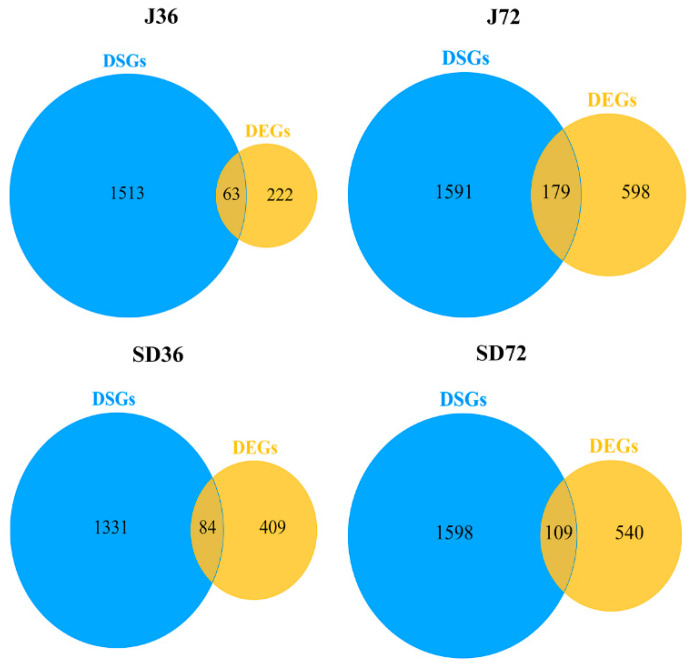
Clustering of DSGs and DEGs in response to *A. alternata* AP infection in apple. Venn diagram showing that the overlap of DSGs and DEGs in J36, J72, SD36 and SD72 groups. Genes with DAS events and FDR < 0.05 were identified as DSGs. Genes with |log_2_FC| ≥ 1 and FDR < 0.01 were identified as DEGs.

To probe the relationship between AS and transcriptional regulation, comparison of the AS and transcriptional levels was preformed among 11,421 AS genes in response to *A. alternata* AP infection. Venn diagrams indicate that there were 1513, 1591, 1331, 1598 DSGs in J36, J72, SD36 and SD72, respectively, which were uniquely responded to *A. alternata* AP at AS level. Alongside, there were 222, 598, 409 and 540 DEGs alternatively spliced, whereas only 63, 179, 84 and 109 AS genes overlapped between DSG and DEG datasets among all four groups (Figure 8). Consequently, AS is an independent regulatory process in response to *A. alternata* AP infection.

**Figure 9:**
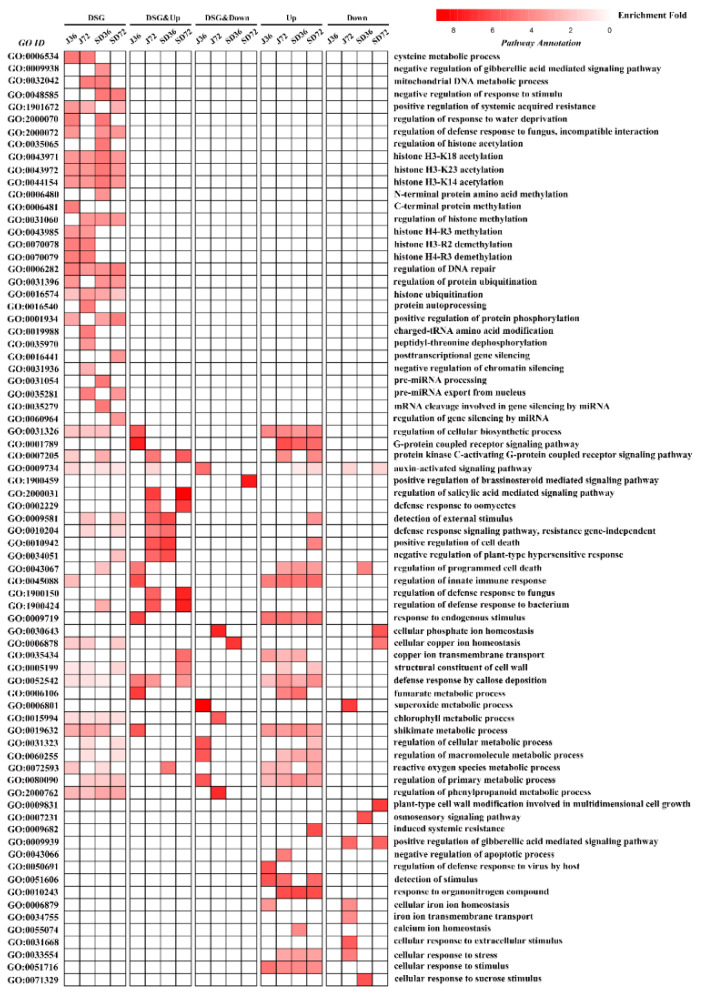
The heatmap showing the GO terms enrichment analysis of DSG-specific, DEG-specific and DSG and DEG-overlapped AS genes. DSG: DSG-specific genes; DSG&Up: AS genes were both differentially spliced and upregulated; DSG&Down: AS genes were both differentially spliced and downregulated; Up: AS genes only were upregulated; Down: AS genes only were downregulated. The color scale represents enrichment folds of different GO terms (FDR < 0.05).

In order to further define the biological functions modulated by AS and/or transcriptional regulation in apple response to *A. alternata* AP infection, we performed the comparative analysis of significantly enriched GO terms on DSG-specific, DEG-specific and DSG and DEG-overlapped genes, respectively. In J36 group, we found that DEG-specific genes were highly associated with ‘detection of stimulus’ and ‘regulation of defense response to virus by host’. DSG and DEG-overlapped genes were significantly enriched in ‘superoxide metabolic process’, ‘G-protein coupled receptor signaling pathway’ and ‘regulation of innate immune response’; while for DSG-specific genes, they were mainly enriched in ‘cysteine metabolic process’, ‘C-terminal protein methylation’, ‘histone H3-R2 demethylation’ and ‘regulation of DNA repair’ pathways. In the J72 group, DEG-specific genes were largely related to ‘response to organonitrogen compound’, ‘cellular response to extracellular stimulus’ and ‘positive regulation of gibberellic acid mediated signaling pathway’. DSG and DEG-overlapped genes were dramatically enriched in ‘cellular phosphate ion homeostasis’, ‘regulation of phenylpropanoid metabolic process’ and ‘regulation of salicylic acid mediated signaling pathway’. As for DSG-specific genes, they were mainly enriched in ‘cysteine metabolic process’, ‘histone H4-R3 demethylation’, ‘protein autoprocessing’ and ‘charged-tRNA amino acid modification’ pathways. In the SD36 group, some GO terms relevant for ‘response to organonitrogen compound’, ‘cellular response to sucrose stimulus’ and ‘osmosensory signaling pathway’ were highly enriched in the DEG-specific genes, whereas various GO terms of ‘cellular copper ion homeostasis’, ‘positive regulation of cell death’ and ‘negative regulation of plant-type hypersensitive response’ were over-represented in DSG and DEG-overlapped genes. Meanwhile, GO terms such as ‘histone H3-K18 acetylation’, ‘pre-miRNA processing’ and ‘mRNA cleavage involved in gene silencing by miRNA’ were mainly enriched in the DSG-specific genes. In the SD72 group, two GO categories linked to ‘plant-type cell wall modification involved in multidimensional cell growth’ and ‘induced systemic resistance’ were largely enriched in the DEG-specific genes. A series of disease resistance-related pathways, such as ‘regulation of salicylic acid mediated signaling pathway’, ‘positive regulation of brassinosteroid mediated signaling pathway’ and ‘regulation of defense response to fungus’, were substantially enriched in DSG and DEG-overlapped genes. Concurrently, the top2 GO categories for the DSG-specific genes were ‘negative regulation of response to stimulus’ and ‘positive regulation of protein phosphorylation’ (Figure 9). Overall, these results unveiled clearly that the AS could be specifically regulated between J and SD cultivars, and preferred to serve as independent roles compared to coordinate with transcriptional regulation in the defensive response to *A*. *alternata* AP infection in apple.

## 3. Discussion

### 3.1. AS Increased the Diversity of Transcripts in Apple

Alternative splicing, as an important post-transcriptional regulatory process, involves in various defense responses. Emerging evidence revealed that AS contributed to the transcriptome diversity whilst enhancing the transcriptome adaption to stresses [25,35,56]. Increasing studies indicate that AS plays important roles in plants regarding coping with pathogen defense [44,46,57,58]. However, little is known about the AS response mechanism in apple defense against *A. alternata* AP infection.

In the present study, there were 11,421 genes that underwent AS in apple, accounting for 40.15% of the multi-exon genes (Table S2). The ratio of AS genes is lower than ~60% in soybean [21] and Arabidopsis [19], but higher than ~10% in wheat [49] and ~24% in rice [37], which reflects the AS divergence among different plants. Interestingly, A3SS was the most frequent type among the five foremost classes, different from universal perception that RI was the most predominant event in plants [17] (Figure 2A,B). The length distribution of RI events had multi-peaks and the average length of intron in RI events was longer than intron in the total genome (Figure S2). These results also markedly differed from prior findings in cotton [35]. In addition, the genes undergoing different types AS events were distributed to different KEGG pathways. Pathways ‘glycosylphosphatidylinositol (GPI)-anchor biosynthesis’ and ‘RNA degradation’ were also mainly enriched in the RI genes, in addition to ‘mRNA surveillance pathway’ enriched in the RI and A3SS genes. Pathways ‘spliceosome’ and ‘circadian rhythm—plant’ were largely enriched in SE and A5SS genes, respectively (Figure 3 and Figure S3). Based on these observations, AS can diversify transcriptome reprogramming, by which it improves the ability of plants to adapt to pathogen stress conditions. Accordingly, the functional significance of AS in response to *A. alternata* AP infection needs to be further investigated in apples.

### 3.2. AS Is an Important Regulation Mechanism of Apple in Response to A. alternata AP Infection

Mounting reports unveiled that some genes underwent specific AS patterns in plants under different stress conditions, between different cultivars or genotypes, even at different tissues [26,56,59]. In Arabidopsis, transcriptome analysis discovered plenty of differentially alternatively spliced isoforms in both resistant and susceptible cultivars after infection with *Pseudomonas syringae* [60] and observed the dynamic changes in AS during *Ralstonia solanacearum* infection [61]. A recent study constructed eleven regulatory modules and obtained six candidate DAS genes linked to pathogen defense response in susceptible and tolerant rapeseed (*Brassica napus* L.) plants after inoculation with *Sclerotinia sclerotiorum* [62]. Intriguingly, specific AS genes exhibited functional differences in the *Pst*-resistant and susceptible plants [49].

Previous studies on apple—*A. alternata* AP interaction focus on transcriptome and proteome levels [7,8,9], while the AS mechanism at post-transcriptional gene regulation level is still ambiguous. Here, there were 7390 DAS events corresponding to 4303 genes, implying that 15.13% of the expressed genes occurred distinct AS modifications in response to *A. alternata* AP infection. Actually, most of the DSGs were not shared at different periods of *A. alternata* AP infection in two cultivars. Statistically, 642 and 585 genes in J and SD cultivar at 36 HPI, and 764 and 742 genes at 72 HPI, were specifically regulated, respectively (Figure 4C,D). It seemed that the AS changes of J cultivar were induced strongly in response to *A. alternata* AP infection compared with SD cultivar, especially at 72 HPI. Importantly, most of the DSGs were mainly enriched in ‘metabolic process’, ‘cell part’, ‘catalytic activity’ and so forth; some of these were even enriched in multiple terms (Figure 5). Thus, AS has complicated and important regulatory function during defense response to *A*. *alternata* AP infection in apples.

### 3.3. Considerable Number of Genes Occurring AS Changes Were Related to Stress Response

To date, expanding findings suggest that gene-provoking AS changes are associated with extensive stresses [63,64,65]. In the study, the frequency of specific DAS events was calculated as 73% and 74% in J and SD, respectively. Serine/threonine protein kinases can phosphorylate serine and threonine residues of proteins and play pivotal roles in the middle and downstream of signal transduction in addition to participating in the initiation of co-stimulatory signals. Previously, *AtEDR1* negatively regulated cell death by mediating ethylene signaling or the ubiquitination pathway [66,67]; *edr1* mutant Arabidopsis plants conferred the powdery mildew (*Golovinomyces cichoracearum*) resistance dependent on the SA or JA signaling pathway [68,69]. In our work, a differential SE event of *MdEDR1-2* increased at two time points in J cultivar in response to *A. alternata* AP infection, but were not altered in SD cultivar, suggesting a potential role in response to *A. alternata* AP infection (Figure 7). The former study revealed that AS could integrate to the nonsense-mediated decay pathway, explaining a complex mechanism in gene regulation [19]. Arabidopsis AFC2 interacted with SR proteins, which was regulated by phosphorylation [70]. SR proteins are well-characterized splicing regulators in higher eukaryotic cells, and some of them interact with the U1-70K protein involved in intron recognition and the early stages of spliceosome assembly in plants [71]. In this research, the *MdAFC2-2* transcript was downregulated in J cultivar during *A. alternata* AP infection compared to the mock-inoculation condition, but upregulated significantly in SD cultivar (Figure 7). Sequence analysis showed that a premature termination codon was introduced in the fourth exon of *MdAFC2-2*, which led to production of the loss-function of truncated protein (Figure 6A). Similarly, the expression of *MdAFC3-4* was strengthened in J cultivar at 72 HPI, while exhibiting an opposite pattern in SD cultivar (Figure 7). It seemed probable that *MdAFC2-2* and *MdAFC3-4* were involved in apple response to *A. alternata* AP and have different functions in two cultivars. However, the specific role of these two transcripts during the infection needs to be further studied.

Transcription factors act as key players in exerting a positive or negative effect on abiotic and biotic stresses [72,73,74,75]. The transcription factor *CmbHLH2* modulated the anthocyanin biosynthesis in chrysanthemum (*Chrysanthemum morifolium*) by the way of alternative splicing [76]. Two alternative splicing transcripts of *ScMYB2* play positive role in drought-induced senescence by mediating the ABA signaling pathway in sugarcane (*Saccharum officinarum*) [77]. Two variants of *AtERF73/HRE1* were involved in hypoxia response and development process [78]. In rice, the lower alternative splicing of *OsbZIP58* increases the heat tolerance via modulating storage material accumulation [79], *OsABI5* variants play a vital role in ABA signaling regulation [80]. Furthermore, *OsWRKY62.1* and *OsWRKY76.1* variants positively regulate the rice resistance to different pathogens [50]. Here, the expression of *MdAGL24-1* and *MdAGL24-2*, as well as *MdWRKY44-1* and *MdWRKY44-2*, showed opposite patterns in apple response to *A. alternata* AP infection, suggesting the potential functional differences of the isoforms during the infection.

### 3.4. AS Provoked Specific Regulatory Functions during A. alternata AP Defensive Response

Previous studies indicate that AS acts as an independent post-transcriptional regulatory mechanism to defend the plants from constantly changing surroundings, with little overlap with transcriptional regulation [32,43]. Additionally, AS and transcriptional modulation collaborate to fight against environmental stress [14,44]. However, the relationship between AS and transcriptional regulation in apple defense against *A. alternata* AP infection remains poorly understood.

In the current study, a comparative analysis of AS genes revealed that the DSG and DEG-overlapped genes were modulated by both AS and transcription, while only a small proportion of DSGs (4% in J36, 10.11% in J72, 5.94% in SD36 and 6.39% in SD72) overlapped with DEGs (Figure 8). The ratios were higher than ~4% in Arabidopsis under salt stress [36], but lower than 28.1% in tea plants under drought conditions [56]. Thus, we speculated that AS and transcriptional modulations were two independent processes during *A. alternata* AP invasion. GO enrichment analysis manifested that various signal transduction, ion homeostasis and metabolic pathways were remarkably enriched in DSG and DEG-overlapped genes in response to *A. alternata* AP infection, including ‘regulation of salicylic acid mediated signaling pathway’, ‘positive regulation of brassinosteroid mediated signaling pathway’, ‘G-protein coupled receptor signaling pathway’, ‘cellular phosphate ion homeostasis’, ‘cellular copper ion homeostasis’, ‘superoxide metabolic process’, and ‘regulation of phenylpropanoid metabolic process’. However, various stress-responsive and histone modification terms were dramatically enriched in DSG-specific genes in response to *A. alternata* AP infection, including ‘negative regulation of response to stimulus’, ‘cysteine metabolic process’, ‘histone demethylation’, and ‘histone acetylation’, which were similar to the results in wheat responsive to drought, heat and their combination stress [25] (Figure 9). It is worth noting that miRNA-mediated post-transcriptional regulation pathways were also significantly over-represented in DSG-specific genes in response to *A. alternata* AP infection, including ‘pre-miRNA processing’, ‘mRNA cleavage involved in gene silencing by miRNA’, ‘regulation of gene silencing by miRNA’. Therefore, AS coupled with miRNA-mediated post-transcriptional gene regulation might play a role during the infection. Collectively, AS preferred to exert specific functions independently, rather than together with transcriptional regulation to defense against *A. alternata* AP infection in apples.

## 4. Materials and Methods

### 4.1. Plant Materials and Fungal Inoculation

Two apple cultivars, ‘Jonathan’ and ‘Starking Delicious’, which are resistant and susceptible to Alternaria blotch disease, respectively, were grafted on *Malus robusta* Rehd. stocks, and grown in a greenhouse. The *A. alternata* AP fungus was cultivated on a potato dextrose agar (PDA) medium incubated at constant temperature incubator for 8~9 days at 24 °C under a dark environment. The young fourth and fifth fully expanded leaves counted from the shoot tips were taken and used to inoculate mycelia together with PDA medium; the inoculation method was carried out according to the protocol described previously [3]. In terms of inoculation assay, the abaxial surface of leaves were inoculated with 4 round mycelium blocks, avoiding the main vein, and mock-inoculation of leaves with PDA medium at each time point as control. Leaves were placed in plastic containers with high humidity then put in climate room at 25 °C under a 16 h light/8 h dark cycle. Samples were collected at 36 and 72 HPI, then immediately frozen in liquid nitrogen and stored at −80 °C for subsequent experiments.

### 4.2. RNA Extraction, RNA-Seq Libraries Construction and Sequencing

Total RNA was extracted from apple leaves using the RNAprep Pure Plant Kit (TIANGEN, Beijing, China) according to the manufacturer’s instruction. The purity and integrity of RNA were tested with the NanoDrop 2000 spectrophotometer (Thermo Fisher Scientific, Waldbroom, MA, USA) and Agilent 2100 Bioanalyzer (Agilent, Santa Clara, CA, USA), respectively. The first cDNA strand was synthesized using random hexamers based on the mRNA template, and then the second cDNA strand was generated by adding buffer, dNTPs, RNase H and DNA polymerase I. The purified double-stranded cDNA was obtained using AMPure XP beads, and then was subjected to terminal repair. A tail and sequencing adaptor were added, and then the fragment size was selected with AMPure XP beads. Sixteen RNA-Seq libraries (each treatment with two biological repeats) were enriched by PCR. The insert size and effective concentration (>2 nM) of cDNA libraries were assessed using Agilent 2100 system and qPCR, respectively. Then, 150 bp paired-end sequencing was performed using Illumina HiSeq platform (Illumina, San Diego, CA, USA). The high-quality clean reads were generated after removing the low-quality reads (those in which more than 50% bases with the quality score ≤ 10), adapter-containing and high-N reads (N radio > 10%). The Q20, Q30 and GC-content of the clean data were calculated. Then, the clean reads were mapped on the apple reference genome (*Malus* × *domestica* GDDH13 Whole Genome v1.1, https://www.rosaceae.org/species/malus/malus_x_domestica/genome_GDDH13_v1.1, accessed on 7 August 2017) by TopHat2 (v2.1.1) software [81] (with the parameters of allowing a maximum of two mismatches) to obtain mapped reads for subsequent bioinformatics analysis.

### 4.3. Detection and Classification of AS Events

The rMATS software (http://rnaseq-mats.sourceforge.net/index.html, accessed on 25 April 2018) was utilized to detect the AS events of apple leaves infected by *A. alternata* AP pathogen and controls. Five major AS events including RI, SE, A3SS, A5SS and MXE were classified from an aligned BAM file and merged reference gtf file, respectively. Using the junction count only quantitative method [82], the DAS events were identified in four comparative groups with the false discovery rate obtained by Benjamini Hochberg-adjusted *p* value (FDR < 0.05). The KEGG enrichment analysis among five different types of AS genes were performed with KOBAS v2.0 [83] and KEGG pathways with FDR < 0.05 were considered significantly enriched. The Venn diagrams were visualized using the online tool Venny 2.1.0 [84] and heatmaps were generated in the R environment (v4.0.5).

### 4.4. Validation of AS Events

Total RNA was isolated according to the method described in RNA sequencing with a slight modification. To avoid sample contamination caused by excessive digestion, the steps for genomic DNA digestion in RNA extraction process were removed, owing to the digestion step also being included in the subsequent cDNA synthesis process. The first strand of cDNA was synthesized in 20 μL reaction volume containing 1 μg total RNA using the Oligo dT Primer and Random 6 mers in the PrimeScript™ RT Reagent Kit with gDNA Eraser (Perfect Real Time) (Takara, Osaka, Japan). Tubulin was used as internal reference gene and PCR products were detected via 1.5% agarose gel. Semi-quantitative polymerase chain reaction (RT-PCR) was performed to examine the AS events using PrimerSTAR GXL DNA Polymerase (Takara, Osaka, Japan). The reaction procedure was as follows: step 1 is initial denaturation, 98 °C for 5 min; step 2 includes denaturation; annealing and extension requires 35 cycles, 98 °C for 10 s, 57 °C for 15 s, and 68 °C for 30 s; step 3 is final extension, 68 °C for 10 min. All the relevant primers were listed in Table S5.

### 4.5. Analysis of Differentially Expressed Genes and Differentially Spliced Genes

For gene expression analysis, fragments per kilobase of transcript per million fragments mapped (FPKM) was calculated based on the gene length and mapped read counts to quantify gene and transcript abundances (FPKM > 1 was record as effective expression) with Cufflinks software (2.2.1) [85]. The differential expression level of genes was analyzed with DESeq2 R package (1.10.1) [86] among four comparative groups and with edgeR (3.0.8) [87] between two samples. Genes with |log_2_FC| ≥ 1 and FDR < 0.01 were defined as differentially expressed genes (DEGs). Genes containing DAS events with FDR < 0.05 were considered as differentially spliced genes (DSGs). GO enrichment analysis was conducted among DSGs with topGO R package (release 2.12) [88]. Moreover, a comparative analysis of the biological functions among all AS genes including DSGs-specific, DEGs-specific and DSGs DEGs-overlapped genes was performed; the significantly enriched GO terms with FDR < 0.05 were visualized in the R environment (v4.0.5).

### 4.6. Analysis of Gene Structure and Protein Domains

The longest open reading frame (ORF) of the transcript sequences were surveyed using bioXM software (v2.7). Gene structures were characterized with online website Gene Structure Display Server 2.0 [89]. The intron retention or exons skipping led to gain or loss of the domains and affected potential function of the proteins, so the Pfam domain pattern of the annotated transcripts and novel transcripts was analyzed with the protein sequences using the HMMER v3.3.2 (http://hmmer.org/, accessed on 13 November 2020) [90] and visualized using TBtools (Toolbox for Biologists v1.0986).

## 5. Conclusions

In the present study, through global analysis of the RNA-Seq data, we investigated the AS diversity between two contrasting cultivars’ defense against *A. alternata* AP infection in apples. We observed that the specific DAS events were significantly induced in the two cultivars at different periods of pathogen infection, especially in J cultivar at 72 HPI. In addition, comparative analysis of the AS genes between DSG and DEG datasets indicates that AS and transcription regulation are two independent processes in apple response to *A. alternata* AP infection. Moreover, the AS provoked specific biological functions, such as involving in the regulation of histone modifications and metabolic processes. Taken together, these results highlighted the critical roles of AS during apple–pathogen interaction and provided a new research direction for resistant apple cultivars breeding.

## Data Availability

The sequencing raw data is available from the NCBI SRA database (PRJNA799397).

## Supplementary Materials

The following supporting information can be downloaded at: https://www.mdpi.com/article/10.3390/ijms232214202/s1.

## Abbreviations

AS	Alternative splicing
DAS	Differential alternative splicing
DSG	Differentially spliced gene
DEG	Differentially expressed gene
FPKM	Fragments per kilobase of transcript per million mapped reads
J	Jonathan
SD	Starking Delicious
HPI	Hours post inoculation
GO	Gene ontology
KEGG	Kyoto Encyclopedia of Genes and Genomes
SE	Skipped exon
RI	Retained intron
A3SS	Alternative 3′ splice sites
A5SS	Alternative 5′ splice sites
MXE	Mutually exclusive exon
*A. alternata* AP	*Alternaria alternata* apple pathotype
*Bgt*	*Blumeria graminis* f. sp. *tritici*
*Pst*	*Puccinia striiformis* f. sp. *Tritici*
*M. oryzae*	*Magnaporthe oryzae*
*Xoo*	*Xanthomonas oryzae* pv *oryzae*
SJs	Splicing junctions
CDS	Coding sequence
5′-UTR	5′ Un-translated region
3′-UTR	3′ Un-translated region
PTC	Premature stop codons
ORF	Open reading frame
NMD	Nonsense-mediated-decay
JA	Jasmonic acid
SA	Salicylic acid
ABA	Abscisic acid
PR	Pathogenesis-related
SR	Serine/arginine-rich
PDA	Potato dextrose agar
