# Supplementary material for: Comparative Analysis of Alternative Splicing in Two Contrasting Apple Cultivars Defense against Alternaria alternata Apple Pathotype Infection
Source: Int J Mol Sci. 2022 Nov 17;23(22):14202. doi: 10.3390/ijms232214202 (PMC9693243; doi:10.3390/ijms232214202)
Supplement: Supplementary file 1 [file ijms-23-14202-s001.zip › Supplementary Figures.pdf]

Supplementary Materials:

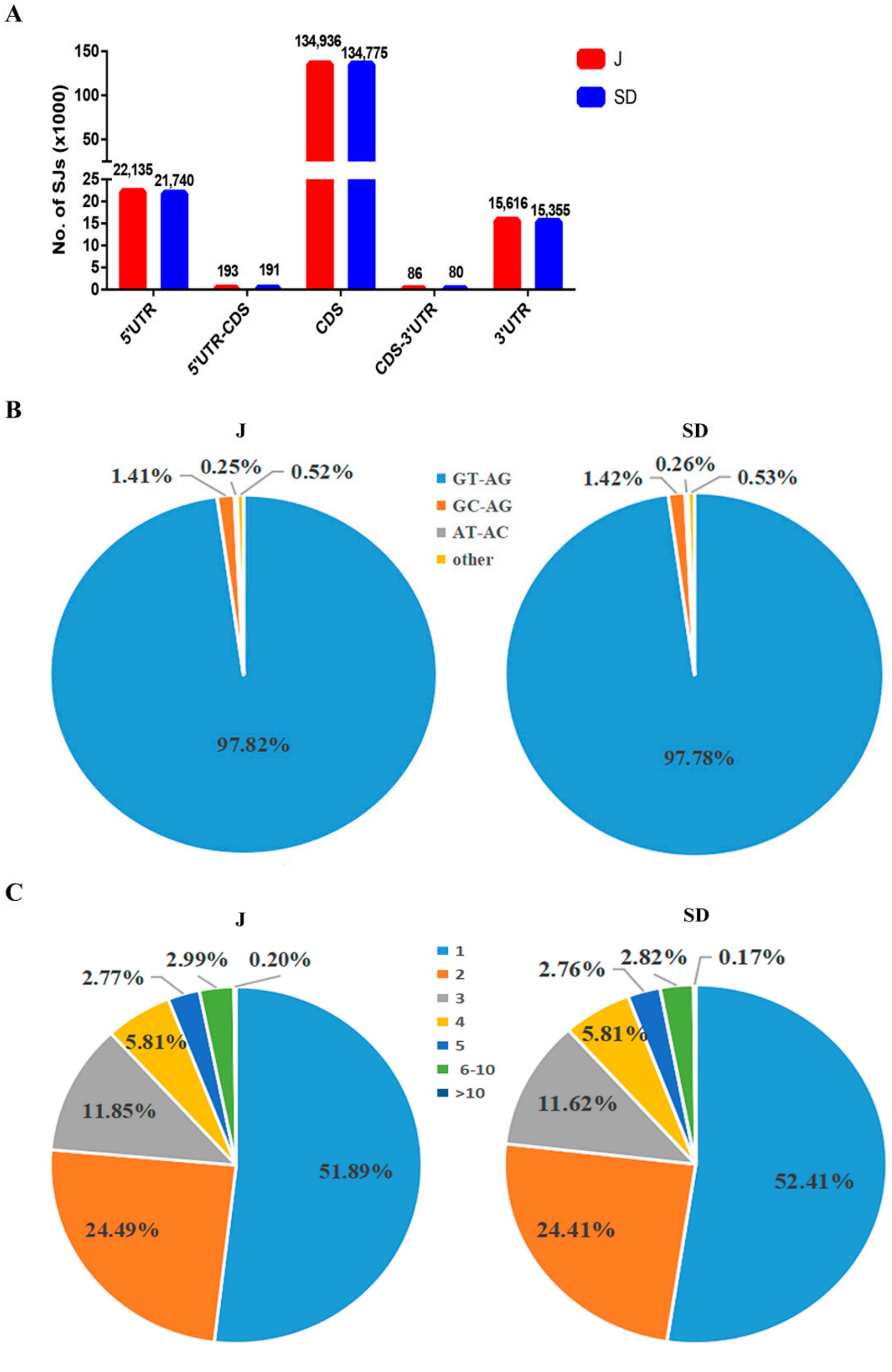

Figure S1. General analysis of alternative splicing in apple. (**A**) Distribution of splicing junctions of all expressed isoforms in the annotated loci. SJ: Splicing Junction. (**B**) Pie charts showing the percentage of the splicing donor-acceptor di-nucleotide utilization among all expressed isoforms. Color scale indicates the types of splicing sites. (**C**) Pie charts showing the percentage of multi-exon genes with different number of isoforms. Color scale indicates the number of isoforms.

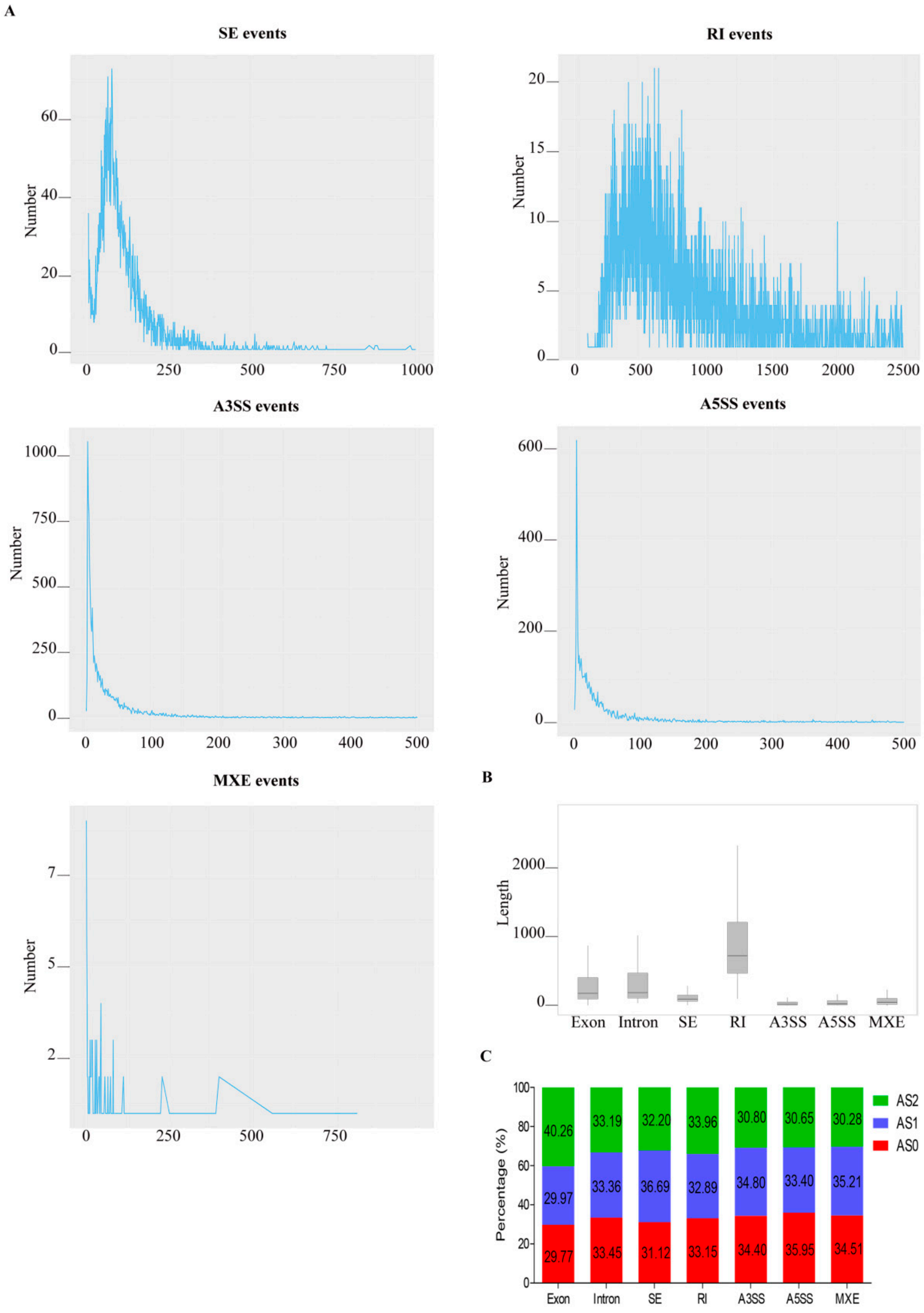

Figure S2. The length size of five major AS events. (**A**) The length distribution of five major AS events. (**B**) Length comparison among exon in whole genome, intron in whole genome and five major AS events. (**C**) Distribution of AS0, AS1and AS2 types and their frequency, respectively. Color scale indicates three categories: AS0, AS1 and AS2. Their frequency were marked within the columns.

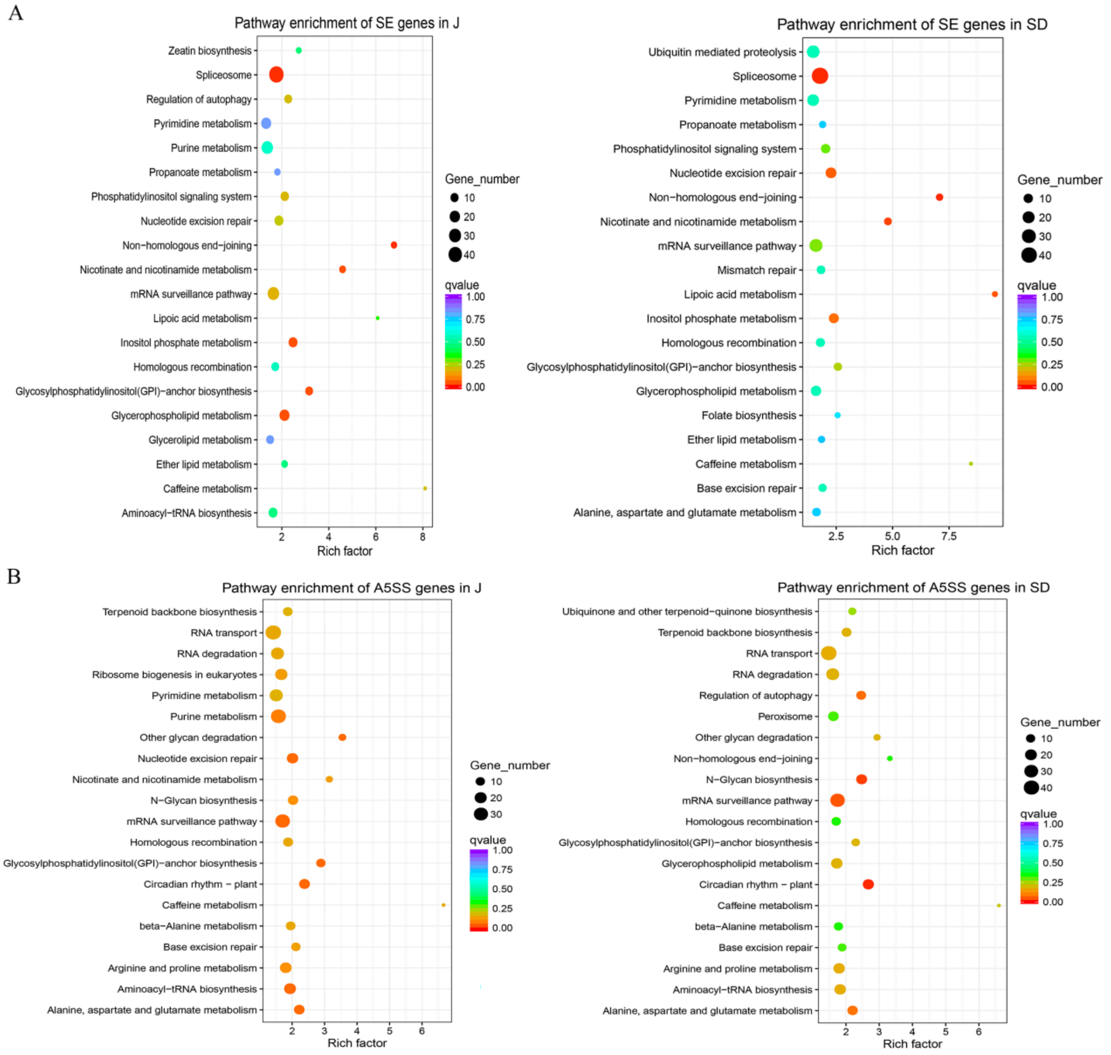

Figure S3. Comparative analysis of enriched KEGG pathways of SE and A5SS genes between J and SD in response to *A. alternata* AP infection. (**A**) Pathway enrichment of SE genes of J and SD in response to *A. alternata* AP infection. (**B**) Pathway enrichment of A5SS genes of J and SD in response to *A. alternata* AP infection. J: overlapped J36 and J72 groups; SD: overlapped SD36 and SD72 groups. The adjusted-*p* value were given with the color bar. The rich factor were noted at the x-axis and gene number were indicated with bubble scale.

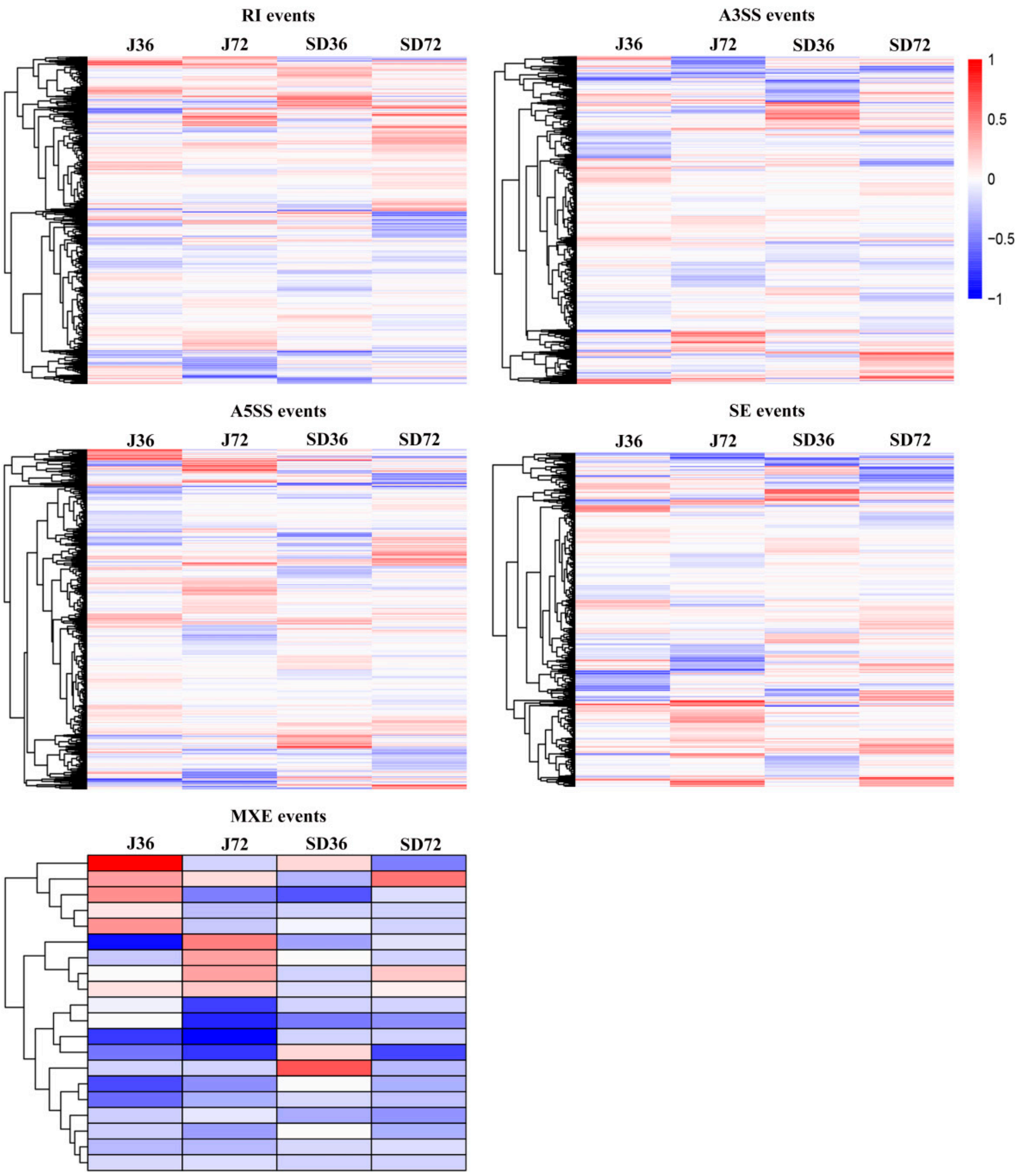

Figure S4. Expression patterns of DAS events in response to *A. alternata* AP infection in apple. Five major DAS events (RI, A3SS, A5SS, SE and MXE) were identified with FDR < 0.05. Red represents the increased and blue represents the decreased AS events in response to *A. alternata* AP infection.

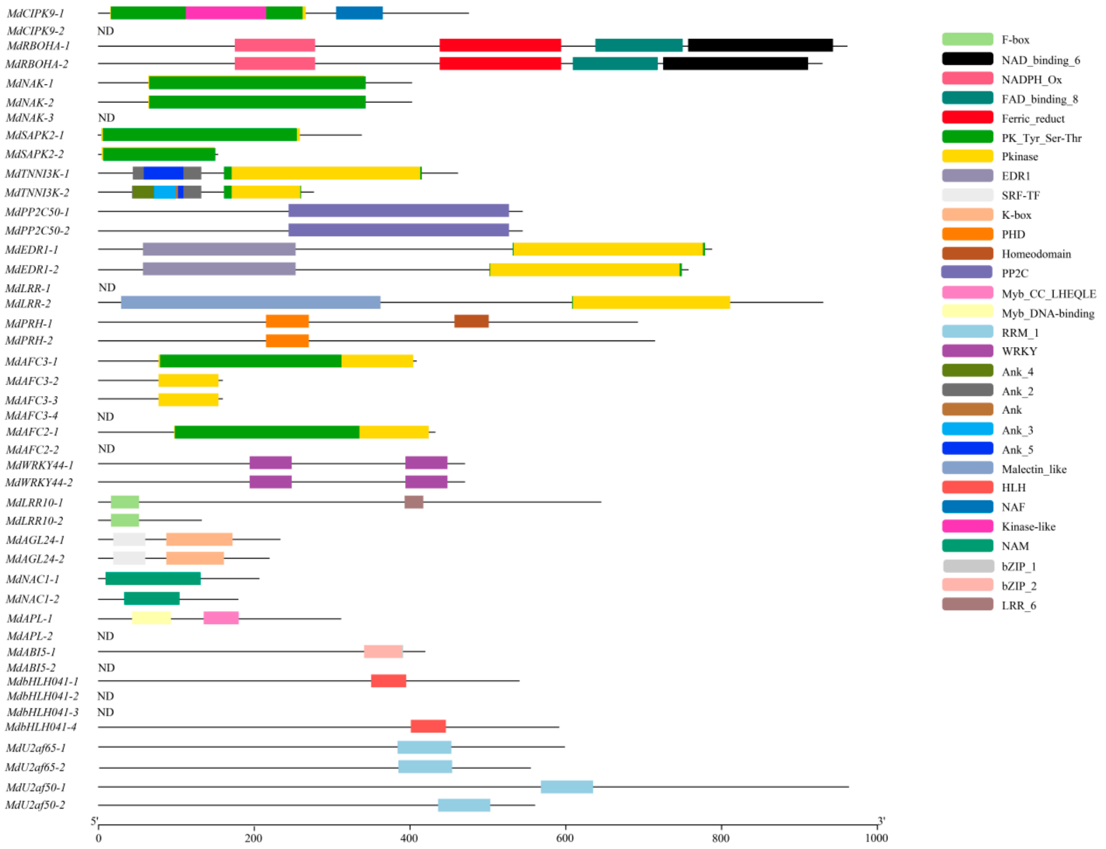

Figure S5. Analysis of protein with a gain or loss of domains due to alternative splicing. For each gene, the annotated transcripts are on the top and the novel transcripts are at the bottom. Color scale indicates the name of domains. ND: No domains.

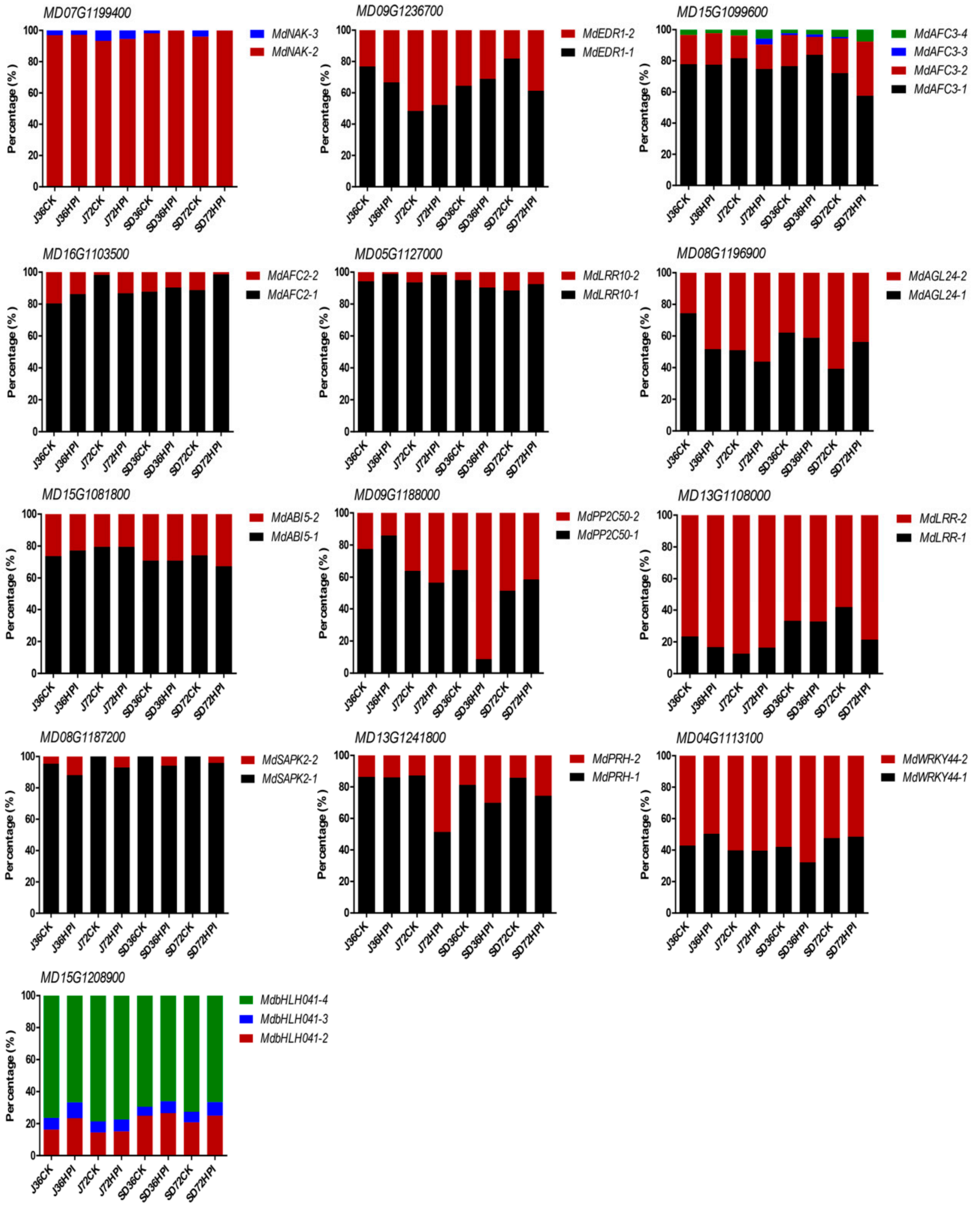

Figure S6. The column charts showing the ratio of pathogen responsive AS events observed in RNA-Seq. The color scale indicates different transcripts in each gene. Gene ID was given at the top of the column charts.
