# Supplementary material for: Comparative Analysis of Alternative Splicing in Two Contrasting Apple Cultivars Defense against Alternaria alternata Apple Pathotype Infection
Source: Int J Mol Sci. 2022 Nov 17;23(22):14202. doi: 10.3390/ijms232214202 (PMC9693243; doi:10.3390/ijms232214202)
Supplement: Supplementary file 1 [file ijms-23-14202-s001.zip › Supplementary Tables.pdf]

Supplementary Materials:

Table S1. Basic information statistics of RNA-Seq data

SampleID	Clean reads	Reads_mapped		Uniq_mapped		Multi_mapped		GC (%)	Q20 (%)	Q30 (%)
		Number	Rate (%)	Number	Rate (%)	Number	Rate (%)			
J36CK1	45,082,848	36,236,275	80.38	36,166,654	99.81	69,621	0.19	47.52	97.86	93.87
J36CK2	45,267,706	36,504,121	80.64	36,437,149	99.82	66,972	0.18	47.58	97.89	93.91
J36HPI1	44,534,866	35,340,321	79.35	35,269,751	99.8	70,570	0.2	47.43	97.86	93.87
J36HPI2	44,812,416	35,787,978	79.86	35,714,300	99.79	73,678	0.21	47.66	97.91	93.99
J72CK1	44,802,036	35,906,025	80.14	35,839,931	99.82	66,094	0.18	47.21	97.89	93.95
J72CK2	45,148,470	36,214,705	80.21	36,148,297	99.82	66,408	0.18	47.18	98.04	94.34
J72HPI1	45,025,800	35,207,017	78.19	35,135,471	99.8	71,546	0.2	47.39	97.85	93.85
J72HPI2	44,530,830	34,737,777	78.01	34,663,166	99.79	74,611	0.21	47.17	97.95	94.09
SD36CK1	43,677,740	34,796,946	79.67	34,729,859	99.81	67,087	0.19	47.58	97.92	94.01
SD36CK2	45,285,140	35,685,868	78.8	35,616,645	99.81	69,223	0.19	47.58	97.84	93.83
SD36HPI1	44,133,228	34,637,368	78.48	34,558,628	99.77	78,740	0.23	47.39	98	94.23
SD36HPI2	44,935,748	35,165,761	78.26	35,089,434	99.78	76,327	0.22	47.4	97.91	93.99
SD72CK1	44,205,606	34,401,382	77.82	34,332,318	99.8	69,064	0.2	47.42	97.61	93.23
SD72CK2	44,171,876	34,700,124	78.56	34,631,400	99.8	68,724	0.2	47.38	97.92	94.01
SD72HPI1	42,053,790	33,121,273	78.76	33,047,853	99.78	73,420	0.22	47.33	97.94	94.07
SD72HPI2	44,911,788	35,290,246	78.58	35,215,616	99.79	74,630	0.21	47.37	97.94	94.08
Average	44,536,243	35,233,324	79.11	35,162,280	99.799375	71,045	0.2	47.41	97.90	93.96

Table S2. The number and percentage of different type AS events

groups	A3SS	RI	A5SS	SE	MXE	AS_event.no
J36	9040<br>(37.04%)	6531<br>(26.76%)	4500<br>(18.44%)	4220<br>(17.29%)	117<br>(0.48%)	24,408
J72	8947<br>(36.48%)	6622<br>(27%)	4485<br>(18.29%)	4359<br>(17.77%)	113<br>(0.46%)	24,526
SD36	9164<br>(37.20%)	6716<br>(27.26%)	4560<br>(18.51%)	4086<br>(16.59%)	107<br>(0.43%)	24,633
SD72	9094<br>(37.04%)	6769<br>(27.57%)	4483<br>(18.26%)	4094<br>(16.68%)	110<br>(0.45%)	24,550
total	13,084<br>(37.65%)	9004<br>(25.91%)	6398<br>(18.41%)	6125<br>(17.62%)	142<br>(0.41%)	34,753

Table S3. The number and percentage of different type AS genes

groups	A3SS	RI	A5SS	SE	MXE	AS_gene.no
J36	5472<br>(58.42%)	3653<br>(39%)	3288<br>(35.11%)	2777<br>(29.65%)	97<br>(1.04%)	9366
J72	5438<br>(58.49%)	3680<br>(39.58%)	3286<br>(35.34%)	2829<br>(30.43%)	97<br>(1.04%)	9297
SD36	5539<br>(58.56%)	3758<br>(39.73%)	3329<br>(35.20%)	2690<br>(28.44%)	94<br>(0.99%)	9458
SD72	5476<br>(58.59%)	3794<br>(40.59%)	3285<br>(35.15%)	2709<br>(28.99%)	94<br>(1.01%)	9346
total	7073<br>(61.93%)	4640<br>(40.63%)	4353<br>(38.11%)	3793<br>(33.21%)	117<br>(1.02%)	11,421

Table S4. The number and percentage of DAS events

groups	RI	A3SS	A5SS	SE	MXE	total
J36	750 (36.93%)	669 (32.94%)	350 (17.23%)	255 (12.56%)	7 (0.34%)	2031 (100%)
J72	919 (37.91%)	779 (32.14%)	405 (16.71%)	312 (12.87%)	9 (0.37%)	2424 (100%)
SD36	654 (35.41%)	606 (32.81%)	309 (16.73%)	277 (15%)	1 (0.05%)	1847 (100%)
SD72	884 (38.49%)	759 (33.04%)	371 (16.15%)	280 (12.19%)	3 (0.13%)	2297 (100%)

Table S5. Primers used in RT-PCR validation of AS events

gene ID	primers	sequences (5´-3´)
MD01G1087300	MdCIPK9-F	GTAAGGAAGGGGCGCAAGAT
	MdCIPK9-R	TTCGTCAAAGAGCTCACCCC
MD06G1093000	MdRBOHA-F	CCAAGCCCTTCAATAGGCTCA
	MdRBOHA-R	TGATTGGGAGAACCTATACTTCCAG
MD07G1199400	MdNAK-F	TCAATTTTTTTCCGCTGTTCG
	MdNAK-R	CATCAACCCACCCCTTAAAGA
MD08G1187200	MdSAPK2-F	AGTCCAGCACCCCGTGTAAA
	MdSAPK2-R	AAGGTGACTCCACAAGACCAAAC

MD09G1155800	MdTNNI3K-F	CATTCAGGGATGAGCTTGCTTT
	MdTNNI3K-R	GCTTGCTCACCCCAAAGTCT
MD09G1188000	MdPP2C50-F	TGAGCTGTCTGAGATCCAGGAA
	MdPP2C50-R	TCCACCCACCAATTCTCTCAA
MD09G1236700	MdEDR1-F	TATTGATGGAGTCGTTGAGTCTG
	MdEDR1-R	CTGTAGCTTGAGGTAGGCATGT
MD13G1108000	MdLRR-F	TTTGCTTTCTGTCATCTGTTGTTTC
	MdLRR-R	GGACTAAATACCTCCTCCTCTCC
MD13G1241800	MdPRH-F	TCAGATACGCAAGTTAGAAGGTCAT
	MdPRH-R	TCTCCTTTGTTCGTGTATTCCTAAG
MD15G1099600	MdAFC3-F	GAGAGTATGTGGCAATCAAGGTA
	MdAFC3-R	ACAGATCACAGGGGTAAGTCCA
MD16G1103500	MdAFC2-F	AAATGGATTACGTGTCGGAGTTC
	MdAFC2-R	TGACCAAAAGTTCCTTCACCAA
MD04G1113100	MdWRKY44-F	GCGTTTCGGTACATGTGCTT
	MdWRKY44-R	TGGCTCAAGATGTGTAAAGTTCA
MD05G1127000	MdLRR10-F	GTAAACACCATTACGCAGATCC
	MdLRR10-R	GAGCAAAGTTATGACGGAGATAGAT
MD08G1196900	MdAGL24-F	AGTAAGGAACTTGAGGATAAGACCC
	MdAGL24-R	CATTATTCAGGTTTTCCAACTCCA
MD08G1222300	MdNAC1-F	ACGAAGAACTTGTAGTCCATTTCCT
	MdNAC1-R	CTTCAATGCCTAATGTTTTCCAG
MD10G1342000	MdAPL-F	AAGAGGGGAGAAAAACAACTCTC
	MdAPL-R	TTGTCCGCATAATAGCCTTTG
MD15G1081800	MdABI5-F	ACCTGCGAACCAGTTGTCAT
	MdABI5-R	CAACTTCTGCTTCCAATTCCAT
MD15G1208900	MdbHLH041-F	CAGGATTCGCTTTCAGGAATAGTAT
	MdbHLH041-R	ATGTTGAAGAGGAGGGAGGAAT
MD02G1151300	MdU2af65-F	CTGAACGGTATTAAAATGGGTGAC
	MdU2af65-R	CATACCAGGATACACGATACAACTC
MD13G1218500	MdRRM1-F	ACCGAGGAAGAGAAGAACCG
	MdRRM1-R	ACTCCACAACAGCTTGACCT

Table S6. General information of AS genes validated by RT-PCR

geneID	gene_ name	Chr	strand	AS_ type	AS_ position	AS_ length	nr_annotation
MD01G1 087300	*MdCIPK9*	Chr 01	+	RI	intron1	488bp	PREDICTED: CBL-interacting serine/threonine-protein kinase 9-like [Malus domestica]
MD06G1 093000	*MdRBOHA*	Chr 06	+	SE	exon7	96bp	PREDICTED: respiratory burst oxidase homolog protein A [Pyrus x

							bretschneideri]
MD07G1 199400	*MdNAK*	Chr 07	+	SE	exon2	92bp	PREDICTED: probable serine/threonine-protein kinase NAK [Malus domestica]
MD08G1 187200	*MdSAPK2*	Chr 08	-	A3SS	intron5	34bp	serine/threonine-protein kinase SAPK2 [Malus domestica]
MD09G1 155800	*MdTNNI3K*	Chr 09	-	SE	exon7	104bp	PREDICTED: serine/threonine-protein kinase TNNI3K [Malus domestica]
MD09G1 188000	*MdPP2C50*	Chr 09	-	RI	intron2	102bp	PREDICTED: probable protein phosphatase 2C 50 [Malus domestica]
MD09G1 236700	*MdEDR1*	Chr 09	+	SE	exon7	90bp	PREDICTED: serine/threonine-protein kinase EDR1-like [Malus domestica]
MD13G1 108000	*MdLRR*	Chr 13	-	RI	intron1	123bp	PREDICTED: probable LRR receptor-like serine/threonine-protein kinase At1g67720 [Malus domestica]
MD13G1 241800	*MdPRH*	Chr 13	-	A5SS	intron8	66bp	PREDICTED: pathogenesis-related homeodomain protein-like isoform X2 [Malus domestica]
MD15G1 099600	*MdAFC3*	Chr 15	+	SE	exon6-8	355bp, 263bp, 115bp	PREDICTED: serine/threonine-protein kinase AFC3 [Pyrus x bretschneideri]
MD16G1 103500	*MdAFC2*	Chr 16	-	SE	exon2	187bp	PREDICTED: serine/threonine-protein kinase AFC2-like isoform X1 [Malus domestica]
MD04G1 113100	*MdWRKY44*	Chr 04	-	A5SS	intron1	111bp	PREDICTED: WRKY transcription factor 44-like [Malus domestica]
MD05G1 127000	*MdLRR10*	Chr 05	-	SE	exon3	94bp	PREDICTED: F-box/LRR-repeat protein 10 [Malus

							domestica]
MD08G1 196900	*MdAGL24*	Chr 08	+	SE	exon6	42bp	PREDICTED: MADS-box protein AGL24-like isoform X1 [Pyrus x bretschneideri]
MD08G1 222300	*MdNAC1*	Chr 08	+	A5SS	intron1	81bp	PREDICTED: LOW QUALITY PROTEIN: NAC transcription factor NAM-1-like [Malus domestica]
MD10G1 342000	*MdAPL*	Chr 10	+	RI	intron1	392bp	PREDICTED: myb family transcription factor APL-like isoform X2 [Malus domestica]
MD15G1 081800	*MdABI5*	Chr 15	-	SE	exon2	110bp	PREDICTED: ABSCISIC ACID-INSENSITIVE 5-like protein 5 isoform X1 [Pyrus x bretschneideri]
MD15G1 208900	*MdbHLH041*	Chr 15	+	RI,A5 SS	intron4	239bp, 157bp	PREDICTED: putative transcription factor bHLH041 [Malus domestica]
MD02G1 151300	*MdU2af65*	Chr 02	-	A3SS	intron12	139bp	PREDICTED: splicing factor U2af large subunit A-like isoform X7 [Malus domestica]
MD13G1 218500	*MdU2af50*	Chr 13	-	A5SS	intron2	327bp	PREDICTED: splicing factor U2af large subunit A-like isoform X1 [Malus domestica]
